# No moderating effect of coping skills on the association between bullying experience and self-esteem: Results from K-CHILD study

**DOI:** 10.3389/fpsyg.2022.1004482

**Published:** 2022-12-14

**Authors:** Yukino Saimon, Satomi Doi, Takeo Fujiwara

**Affiliations:** ^1^Department of Global Health Promotion, Tokyo Medical and Dental University, Tokyo, Japan; ^2^Research Fellow of Japan Society for the Promotion of Science, Tokyo, Japan

**Keywords:** bullying experience, self-esteem, coping skill, Japan, adolescent

## Abstract

**Introduction:**

Few studies have investigated the moderating effect of coping skills on the association between bullying experience and low self-esteem. The aim of this study was to examine whether coping skills have a moderating effect on the association between bullying experience and self-esteem among Japanese students.

**Methods:**

Data from the population-based Kochi Child Health Impact of Living Difficulty (K-CHILD) study conducted in 2016 were analyzed. Participants included fifth-and eighth-grade students living in Kochi Prefecture, Japan. A questionnaire for the students (*n* = 5,991) assessed the bullying experience, self-esteem (the Japanese Edition of the Harter’s Perceived Competence Scale for Children), and coping skills that comprised six types (The shortened version of coping skills for elementary school children). Multivariate linear regression analyses were conducted to examine the association between bullying experience and self-esteem and then the moderating effects of six types of coping as interaction terms on the association were considered.

**Results:**

Bullying experience was inversely associated with self-esteem. All six types of coping did not moderate the relationship between bullying experience and low self-esteem even after adjusting for cofounders (all *P* for interaction > 0.15).

**Conclusion:**

Coping skills did not moderate the association between bullying experience and self-esteem, suggesting that intervention to boost coping skills to mitigate the adverse effect of bullying experience may not be promising.

## Introduction

Bullying is a notable social problem at school and the workplace globally. The 2018 Program for International Student Assessment reported that 23% of students reported being bullied at least a few times a month on average across OECD countries. Although the definition of bullying may vary by research, bullying generally involves (1) intentionality, (2) repetition, and (3) a clear power imbalance between perpetrator and victim (Olweus, 1993). Regardless of the varied components of bullying, the outcome of bullying for victims is often the deterioration of mental health, such as depression, anxiety, or suicidality (Azúa Fuentes et al., 2020). The global school-based Student Health Survey conducted in Asia, Africa, and South America (2009–2017) showed that children who had bullying victimization were 2.8 times more likely to attempt suicide (Smith et al., 2021). In Japan, similarly, a study that investigated the risk factors for suicidality reported that school bullying had a high odds ratio (OR) in junior high students (OR: 3.1, 95% confidence interval (CI): 2.1–4.4) and high school students (OR: 3.6, 95% CI: 2.5–5.3) (Nagamitsu et al., 2020).

Low self-esteem induced by bullying (van Geel et al., 2018) is the main cause of serious mental illnesses in victims. A previous study found that self-esteem mediated the association between bullying victimization and depression (Zhong et al., 2021). Furthermore, low self-esteem in childhood and early adolescence had an influence on depression, anxiety, and internalizing disorders in later adolescence and adulthood (Sowislo and Orth, 2013; Keane and Loades, 2017). Moreover, students who reported having low self-esteem were more likely to declare poorer self-rated health afterward (Arsandaux et al., 2019). Eventually, low self-esteem was closely related to suicidality (Overholser et al., 1995; McGee et al., 2001; Manani and Sharma, 2016).

Several studies have discussed the effect modifiers that can attenuate the association between bullying experience and low self-esteem, such as social support, friendship, and characteristics of victims like mindfulness and forgiveness (Zhou et al., 2017; Barcaccia et al., 2018). Among them, specific coping ways can be a possible moderator of the association between being bullied and low self-esteem. In general, the coping style includes emotion-focused coping which aims to manage emotions associated with the stressor and problem-focused coping which aims to tackle the problems (Folkman and Lazarus, 1980). Among children and adolescents, problem-focused coping is positively associated with self-esteem (Cong et al., 2021). Another study that assessed workplace bullying showed that victims who chose to seek help or assertiveness as a coping style (i.e., problem-focused coping) were more likely to keep high self-esteem scores in comparison with victims who chose other coping styles, while avoidance of coping style (i.e., emotion-focused coping) deteriorated the influence of being bullied on self-esteem (Bernstein and Trimm, 2016). However, to the best of our knowledge, no study investigates the moderating effect of the association between bullying experience and self-esteem among school children.

In a study that compared college students in Japan and those in the United States, it was revealed that the prevalence of students who had engaged in bullying as perpetrators was significantly higher in Japan than in the United States (17.8 vs. 11.7%) (Kobayashi and Farrington, 2020). In addition, as Japanese tend to have lower self-esteem and are more self-critical than their Western counterparts (Heine et al., 1999; Heine and Hamamura, 2007), Japan is a suitable setting to assess the modifiable factors that mitigate the association between bullying experience and low self-esteem.

The aim of this study was to investigate whether bullying experience affects self-esteem and whether coping skills have a moderation effect on the association or not. This study hypothesized that (1) bullying experience is associated with low self-esteem and (2) coping skills moderate the association between bullying experience and low self-esteem.

## Materials and methods

### Participants

We analyzed data from the Kochi Child Health Impact of Living Difficulty (K-CHILD) study conducted in 2016 (Doi et al., 2020). TF designed the K-CHILD study in consultation with Kochi Prefecture. The objective of this study was to assess the living and health conditions of elementary school, junior high school, and high school students and their parents in Kochi Prefecture, Japan. Kochi Prefecture has a population of 690,211 and 317,822 households as of September 2020. In Kochi, 2.55% of households receive public assistance as of August 2021, and the rate is the fourth highest of 47 Prefectures in Japan. Kochi Prefecture has bolstered its policies to improve the physical and mental health of children from socioeconomically disadvantaged backgrounds.

The participants of the K-CHILD study were students in the first and fifth grades in all elementary schools, students in the eighth grade in all junior high schools, and students in the eleventh grade in all high schools. K-CHILD targeted all public, private, and special needs schools in the prefecture, except for correspondence high schools and one special needs school. The Office of Children and Family Services in Kochi Prefecture mailed questionnaires to each school. The anonymous and self-answered questionnaires for children and their caregivers were distributed to children by their teachers. The caregivers’ version of the questionnaires was brought home to the caregivers by their children. The total number of children who received the questionnaires was 23,750, of which 5,460 were in the first grade, 5,764 in the fifth grade, 6,192 in the eighth grade, and 6,334 in the eleventh grade. A total of 14,539 completed children’s and caregivers’ questionnaires were returned to the Office of Children and Family Services in Kochi City (the biggest city in the prefecture) by mail anonymously or to the schools in-person in anonymous envelopes (overall response rate: 61.2%). Approximately 9,108 questionnaires were distributed to participants, and 3,517 of them were returned by postal mail, so the response rate was 38.6%; 14,642 questionnaires were distributed to participants, and 11,022 of them were returned to the schools in person, so the response rate was 75.3%. Notably, 102 child–caregiver pairs returned questionnaires that were blank. Hence, the sum of valid responses was 14,437.

**FIGURE 1:**
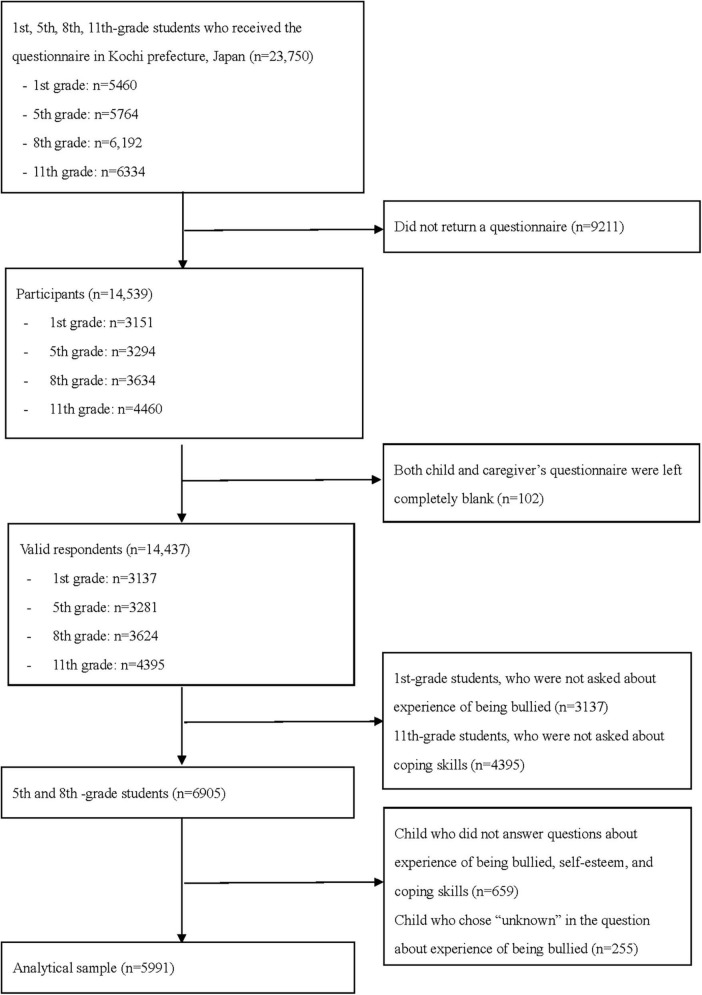
Requirement flow chart.

In our study, the exclusion criteria were as follows: (1) the 1st-grade students because they were not asked about bullying experience; (2) the 11th-grade students because they were not asked about coping skills; (3) children who did not answer questions about bullying experience, self-esteem, and coping skills; and (4) children who chose “unknown” in the question about bullying experience. We excluded from our analysis the first-grade students who were not asked about bullying experience (*n* = 3,137) and the eleventh-grade students who were not asked about their coping skills (*n* = 4,395). Furthermore, we excluded the respondents who did not answer any of our main variables, such as bullying experience, self-esteem, or coping skills, and respondents who answered unknown in the questionnaire on bullying experience. Finally, our analytical sample consisted of 5,991 pairs in the fifth (*n* = 2,906) and eighth grades (*n* = 3,085) (Figure 1).

### Measurements

#### Bullying experience

The question “Have you ever been bullied?” was asked, and the answer could be 1 (often), 2 (sometimes), 3 (occasionally), 4 (never), or 5 (unknown). In our study, we used the respondents of 1–4 as a continuous variable, and 5 was categorized as missing because it was not an ordinal variable. To avoid the burden of responding to being bullied experience, we did not assess the types of bullying experiences.

#### Coping skills

Children’s coping skills were calculated by using 12 items on a scale of the shortened version of coping skills for Japanese elementary school children (Otake et al., 2001). They were asked about the frequency of their way of coping when they were confronted by troubles. The 12 means of coping are as follows: (1) asking someone how to cope with troubles, (2) asking for help, (3) trying to change oneself, (4) trying to realize the causes of troubles, (5) being alone, (6) crying alone, (7) yelling in a loud voice, (8) complaining to someone, (9) quitting to think deeply, (10) giving up doing anything, (11) playing games, and (12) playing with friends. The Cronbach’s alpha in this study was 0.579. Children rated these items as 1 (rarely), 2 (occasionally), 3 (sometimes), and 4 (often) and then six scores of coping styles were calculated by adding two corresponding questions out of (1) to (12): (a) asking for help as a coping type was calculated by adding two scores of (1) and (2), (b) solving problem was from (3) to (4), (c) emotional avoidance was from (5) to (6), (d) behavioral avoidance was from (7) to (8), (e) cognitive avoidance was from (9) to (10), and (f) diversion was from (11) to (12). Based on previous studies (Folkman and Lazarus, 1980; Machmutow et al., 2012), problem-focused coping includes (a) asking for help and (b) solving problems, while emotion-focused (avoidance) coping includes (c) emotional avoidance, (d) behavioral avoidance, (e) cognitive avoidance, and (f) diversion. When one of the two corresponding questions decided a coping style was missing, we used another score for imputation. When both two corresponding questions were not answered, we dealt with the sample as missing.

#### Self-esteem

Children’s self-esteem was estimated using the subscale of the self-esteem of the Japanese Edition of Harter’s Perceived Competence Scale for Children (Sakurai, 1992). It consists of nine items: “Are you confident in yourself?,” “Do you think you can manage most things better than anyone else?,” “Do you think you have many values that you are proud of?,” “Do you feel you would succeed if you tried anything?,” “Are you satisfied with what you are now?,” “Do you think you can become a great person?,” “Do you think you have many values for society?,” “Are you able to express your opinion with confidence?,” and “Do you think you have many good points?.” The children answered each item with a scale of 0 (no), 1 (rather no), 2 (rather yes), and 3 (yes). A higher total score means a higher level of self-esteem. The Cronbach’s alpha was 0.86 in this study.

#### Covariates

The caregivers were asked about marital status (“married,” “unmarried,” “divorced,” or “widowed”), maternal and paternal education level (“junior high school,” “dropout high school,” “high school,” “technical college,” “junior college,” “dropout college education,” “college education,” “graduate college,” “other,” or “unknown”), annual household income (in JPY1 million units; JPY110 equivalent to USD1), lack of daily necessities (“yes” or “no”), and the experience of relocation (“yes” or “no”). As for children’s sex, height, and weight, the eighth grade answered the questions themselves while caregivers of the fifth grade answered on behalf of them. Children’s body mass index (BMI) was calculated by WHO Growth reference 5–19 years and categorized into “underweight,” “normal,” and “overweight” (de Onis et al., 2007).

School location was categorized with municipalities where the schools that joined our study were located (Table 1). As for scores of coping ways, the category of diversion had the highest mean, while behavioral avoidance had the lowest mean. Details are shown in Table 2.

**TABLE 1:** Descriptive characteristics of study participants.

				Experience of bullying							
		Total		Never (4675, 78.0%)		Occasionally (638, 10.6%)		Sometimes (510, 8.5%)		Often (168, 2.8%)	
							
		*n* or mean	% or SD	*n* or mean	% or SD	*n* or mean	% or SD	*n* or mean	% or SD	*n* or mean	% or SD
Child sex	Boy	2750	45.9	2072	44.3	334	52.4	262	51.4	82	48.8
	Girl	3100	51.7	2485	53.2	291	45.6	242	47.5	82	48.8
	Missing	141	2.4	118	2.5	13	2.0	6	1.2	4	2.4
Grade	5	2906	48.5	2160	46.2	351	55.0	301	59.0	94	56.0
	8	3085	51.5	2515	53.8	287	45.0	209	41.0	74	44.1
BMI	Underweight (< −1SD)	272	4.5	218	4.7	29	4.6	21	4.1	4	2.4
	Normal (≧ −1SD, < 1SD)	3071	51.3	2469	52.8	322	50.5	217	42.6	63	37.5
	Overweight (≧ 1SD)	1508	25.2	1126	24.1	168	26.3	156	30.6	58	34.5
	Missing	1140	19.0	862	18.4	119	18.7	116	22.8	43	25.6
Paternal age	Mean	44.61	5.92	44.59	5.82	44.69	6.28	44.96	6.28	44.04	6.27
Maternal age	Mean	42.63	4.90	42.69	4.86	42.46	5.10	42.39	5.15	42.31	4.68
Marital status	Married	4948	82.6	3868	82.7	533	83.5	418	82.0	129	76.8
	Unmarried/ Divorced/Widowed	981	16.4	756	16.2	101	15.8	85	16.7	39	23.2
	Missing	62	1.0	51	1.1	4	0.6	7	1.4	0	0.0
Paternal education	High school or less	2425	40.5	1904	40.7	238	37.3	215	42.2	68	40.5
	Some college	1055	17.6	831	17.8	124	19.4	74	14.5	26	15.5
	College or more	1474	24.6	1143	24.5	169	26.5	124	24.3	38	22.6
	Other/Unknown	24	0.4	14	0.3	4	0.6	5	1.0	1	0.6
	Missing	1013	16.9	783	16.8	103	16.1	92	18.0	35	20.8
Maternal education	High school or less	2023	33.8	1595	34.1	193	30.3	173	33.9	62	36.9
	Some college	2625	43.8	2054	43.9	279	43.7	218	42.8	74	44.1
	College or more	934	15.6	712	15.2	115	18.0	86	16.9	21	12.5
	Other/Unknown	28	0.5	18	0.4	6	0.9	3	0.6	1	0.6
	Missing	381	6.4	296	6.3	45	7.1	30	5.9	10	6.0
Household income	< 3 million yen	1097	18.3	833	17.8	116	18.2	106	20.8	42	25.0
	≧ 3 million yen	3815	63.7	2987	63.9	405	63.5	323	63.3	100	59.5
	Missing	1079	18.0	855	18.3	117	18.3	81	15.9	26	15.5
Experience of financial difficulties	No	5009	83.6	3950	84.5	529	82.9	401	78.6	129	76.8
	Yes	798	13.3	578	12.4	90	14.1	96	18.8	34	20.2
	Missing	184	3.1	147	3.1	19	3.0	13	2.6	5	3.0
Lack of daily necessities	No	3695	61.7	2926	62.6	384	60.2	299	58.6	86	51.2
	Yes	1096	18.3	802	17.2	131	20.5	118	23.1	45	26.8
	Missing	1200	20.0	947	20.3	123	19.3	93	18.2	37	22.0
Experience of relocation	No	1537	25.7	1205	25.8	153	24.0	134	26.3	45	26.8
	Yes	1469	24.5	1109	23.7	178	27.9	137	26.9	45	26.8
	Missing	2985	49.8	2361	50.5	307	48.1	239	46.9	78	46.4

**TABLE 2:** Distributions of self-esteem and coping scores.

				Experience of bullying							
		Total		Never		Occasionally		Sometimes		Often	
		Mean	SD	Mean	SD	Mean	SD	Mean	SD	Mean	SD
Self-esteem	Range: (0–27)	14.63	6.00	15.12	5.88	13.82	5.83	12.44	6.19	10.95	6.33
Types of coping	Asking for help	3.46	1.77	3.46	1.77	3.51	1.72	3.48	1.79	3.34	1.99
	Solving problem	3.99	1.90	3.98	1.90	4.13	1.76	3.93	1.97	3.81	2.12
	Emotional avoidance	3.64	1.90	3.55	1.85	3.90	1.90	4.06	2.08	4.19	2.38
	Behavioral avoidance	3.21	1.47	3.17	1.44	3.35	1.48	3.38	1.57	3.35	1.79
	Cognitive avoidance	3.47	2.03	3.44	2.02	3.69	1.95	3.57	2.13	3.45	2.32
	Diversion	4.02	2.13	4.01	2.12	4.21	2.06	4.06	2.20	3.66	2.33

### Statistical analysis

First, a multivariate linear regression analysis was conducted to examine the association between bullying experience and self-esteem. The independent variable was the frequency of being bullied (i.e., never, occasionally, sometimes, and often). The dependent variable was the total score of self-esteem. Then, the child’s sex (“male” or “female”), BMI (“underweight,” “normal,” or “overweight”), grade (“5th” or “8th”), lack of daily necessities (“yes” or “no”), the experience of financial difficulties (“yes” or “no”), household income, mothers’ educational background, fathers’ educational background, experience of relocation, parents’ marital status, and school location were added as confounders.

Then, we also performed a multivariate linear regression analysis to examine the moderating effect of six types of coping as an interaction term (i.e., victimization * each type of coping) on the association between victimization and self-esteem. These analyses were weighted for response rates of each municipality (i.e., probability weight). A significant level in this study is 5%. All analyses were conducted by using STATA version 15.0 SE (StataCorp. 2017, StataCorp LLC, College Station, TX, USA).

## Results

### Distribution of characteristics

Table 1 shows the distribution of characteristics by frequency of bullying experience. Among all participants, the proportion of the child’s sex and school grade is nearly equal. About 25% of students are overweight. Over half of maternal and paternal age was concentrated between 40 and 49 years, and over 80% of mothers were married. Nearly 60% of fathers had an educational background of some college or less, and over 70% of mothers had a background of some college or less. Nearly 20% had low household income (< JPY3 million), over 10% of caregivers reported that they have experienced financial difficulties, and over 15% of them experienced a lack of daily necessities. Nearly 25% reported experience of relocation.

Among the participants, 4,675 (78.0%) reported they had never been bullied, 638 (10.6%) reported they had occasionally been bullied, 510 (8.5%) reported they had sometimes been bullied, and 168 (2.8%) reported they had often been bullied.

### Association between bullying experience and self-esteem

Table 3 shows the association between the frequency of bullying experience and self-esteem. According to the analysis, the more frequently children were bullied, the lower their self-esteem score became even after adjusting for confounders (*P* for trend: < 0.001); those who had occasionally been bullied (coefficient: −1.60; 95%CI: −2.09, −1.12); those who had sometimes been bullied (coefficient: −2.99; 95%CI: −3.54, −2.44); and those who had often been bullied (coefficient: −4.20; 95%CI: −5.20, −3.19).

**TABLE 3:** Association between the frequency of bullying experiences and self-esteem score.

		Crude	Adjusted
			
		Coefficient (95% CI)	Coefficient (95% CI)
Frequency of bullying experiences	Never	Ref	Ref
	Occasionally	−1.30 (−1.79, −0.81)	−1.60 (−2.09, −1.12)
	Sometimes	−2.68 (−3.22, −2.14)	−2.99 (−3.54, −2.44)
	Often	−4.16 (−5.07, −3.25)	−4.20 (−5.20, −3.19)
	*P* for trend	< 0.001	< 0.001

Adjusted for sex, BMI, grade, daily necessities, experience of financial difficulties, household income, mothers’ educational background, fathers’ educational background, experience of relocation, parents’ marital status, and school location.

### The moderating effect of various coping styles on the relationship between bullying experience and self-esteem

Table 4 indicates that the interaction term of the bullying victimization score as a continuous variable and the score of each coping way as a continuous variable did not make a significant difference in self-esteem score (*P* > 0.1 in any type of coping). There was no significant influence of coping ways on the association between bullying experience and levels of self-esteem.

**TABLE 4:** Effect of each coping skill as a moderator on the association between bullying experience and self-esteem score.

Type of coping	Crude		Adjusted	
	Coefficient (95% CI)	*P*-value	Coefficient (95% CI)	*P*-value
Asking for help	0.082(−0.024,0.19)	0.13	0.064(−0.048,0.18)	0.27
Solving problem	0.036(−0.067,0.14)	0.50	0.040(−0.071,0.15)	0.48
Emotional avoidance	0.024(−0.075,0.12)	0.63	0.048(−0.059,0.15)	0.38
Behavioral avoidance	0.050(−0.072,0.17)	0.43	0.044(−0.078,0.16)	0.48
Cognitive avoidance	−0.043(−0.16,0.071)	0.50	−0.013(−0.14,0.11)	0.84
Diversion	−0.036(−0.14,0.064)	0.48	−0.048(−0.15,0.058)	0.38

Adjusted for sex, BMI (body mass index), grade, daily necessities, experience of financial difficulties, household income, mothers’ educational background, fathers’ educational background, experience of relocation, parents’ marital status, and school location.

## Discussion

In our study, we found that there was a dose–response association between bullying victimization and self-esteem; victims who had been bullied more frequently were likely to have lower self-esteem. Furthermore, any type of coping did not moderate the association, that is, victims’ self-esteem was consistently deteriorated by bullying experience, regardless of coping skills.

Our finding on the association between bullying victimization and low self-esteem was consistent with the hypothesis. Based on the diathesis-stress model, bullying victimization, or negative evaluations from others, can activate cognitive vulnerabilities (Swearer and Hymel, 2015). Furthermore, the time perspective can explain the mechanism of the association between bullying experience and low self-esteem. Time perspective refers to individual thoughts and feelings about their past, present, and future (Zimbardo and Boyd, 2015), which is developed and varies in adolescence (Mello, 2019). Bullying experiences may cause negative feelings about the past, present, and future, and more frequency to think about the past, which leads to lower self-esteem according to previous findings (Moon and Mello, 2021).

The lack of moderating effect on the association between bullying experience and self-esteem was inconsistent with previous studies that showed the interaction effect of coping styles on the association between bullying experience and mental health. Our hypothesis that coping skills moderate the association between bullying experience and low self-esteem was not supported. Coping self-efficacy and emotion dysregulation had a mediation effect on the association between cyber victimization and internalizing difficulties (Trompeter et al., 2018), and coping partially mediates the association between appearance−related bullying problems and self−esteem among young students in Australia (Lodge and Feldman, 2007). The way that one seeks help and assertiveness moderated the impact of bullying victimization on individual well-being including self-esteem, whereas avoidance had a mediation effect on the relationship on workplace bullying in South Africa (Bernstein and Trimm, 2016). Although these studies reported that specific ways of coping had moderating or mediating effects on the association between bullying victimization and self-esteem, our study did not find any moderating effects of coping ways. We consider this inconsistency to be attributed to the specific characteristics of the Japanese culture, that is, collectivism. Compared with other cultures, the Japanese are more likely to think that individuals should behave in a similar way to others. Although individualism had been prevalent as modernization progressed around the world, collectivistic values remained in Japan in some aspects of family and friendship (Hamamura, 2012). Furthermore, the number of Japanese who selected “respect individual rights” as an important moral principle had decreased. Thus, a coping strategy may not protect from deterioration of mental health because students who behave based on a coping strategy will be seen as outliers, which would lead to further isolation from peers. As a result, bullying in Japan would have a tragic impact on victims, regardless of their coping capability.

According to our findings, a strategy that promotes children’s coping skills may not be helpful to mitigate the adverse impacts of bullying experiences, at least among Japanese children. Rather than the strategy to change the children’s behaviors, it may be important to create a support system around the children. A previous study showed that social support mitigates the adverse impacts of bullying experiences (Machmutow et al., 2012; Mishna et al., 2016). An approach to foster the relationship between children and parents, teachers, or other adults and to create a third place for children may be effective to buffer the impacts of bullying and prevent bullying on self-esteem (Fujiwara et al., 2020).

This study has several limitations. First, it is a cross-sectional study. We could not evaluate the causality between bullying victimization and low self-esteem. Some studies mentioned reverse causality, that is, low self-esteem in children led to a higher likelihood of being victims of school bullying (Brito and Oliveira, 2013; Silva et al., 2020). Second, the data of the K-CHILD study do not reflect the situation throughout Japan. Furthermore, the response rate in Kochi City was lower than in other municipalities. Therefore, this study cannot be generalized to the whole of Japan. Third, the experience of being bullied is a self-rated question, so some students might not remember correctly, and some students might not want to answer the question to keep their experience a secret. Besides, some students answered unknown questions on bullying (4.21% of 6,606 students), but we have excluded them from this study. Therefore, there is a possibility that students who had been bullied and kept it secret were excluded. Fourth, we could not assess the type of bullying, intentionality, repetition, and power imbalance due to using one question “Have you ever been bullied?” in this study. Especially, the power imbalance between the bully and victim is an important aspect because it helps to distinguish between bullying and other aggressive behaviors (Aalsma and Brown, 2008). Although it remains difficult to define bullying and a detailed assessment of bullying has risks in the school (Olweus, 1993; Basilici et al., 2022), we need to assess the multiple aspects of bullying experiences in a further study.

The most significant point in the settings of education is to eradicate bullying itself. Bullying has a major and lasting impact on students’ mental health and suicidality. Therefore, if adults noticed or encountered victimization, they should not ask victims to cope themselves but instead, make an effort not to repeat the bullying.

## Data availability statement

The datasets presented in this article are not readily available because of the restriction of IRB. Requests to access the datasets should be directed to TF, fujiwara.hlth@tmd.ac.jp.

## Ethics statement

The studies involving human participants were reviewed and approved by Ethics Committee of the Tokyo Medical and Dental University (M2017-243). Written informed consent from the participants’ legal guardian/next of kin was not required to participate in this study in accordance with the national legislation and the institutional requirements.

## Author contributions

TF designed the study. TF and SD managed the administration of the study, including the ethical review process, and provided critical comments on the manuscript related to the intellectual content. YS analyzed data and drafted the manuscript. All authors have read and approved the final manuscript.

## References

[B1] Aalsma M. C. Brown J. R. (2008). What is bullying? *J. Adolescent Health* 43 101–102. 10.1016/j.jadohealth.2008.06.001 18639781

[B2] Arsandaux J. Michel G. Tournier M. Tzourio C. Galéra C. (2019). Is self-esteem associated with self-rated health among French college students? a longitudinal epidemiological study: the i-Share cohort. *BMJ Open* 9:e024500. 10.1136/bmjopen-2018-024500 31167858 PMC6561426

[B3] Azúa Fuentes E. Rojas Carvallo P. Ruiz Poblete S. (2020). [Bullying as a risk factor for depression and suicide]. *Rev. Chil. Pediatr.* 91 432–439. 10.32641/rchped.v91i3.1230 32730526

[B4] Barcaccia B. Pallini S. Baiocco R. Salvati M. Saliani A. M. Schneider B. H. (2018). Forgiveness and friendship protect adolescent victims of bullying from emotional maladjustment. *Psicothema* 30 427–433. 30353845 10.7334/psicothema2018.11

[B5] Basilici M. C. Palladino B. E. Menesini E. (2022). Ethnic diversity and bullying in school: a systematic review. *Aggress. Violent Behav.* 65:101762. 10.1016/j.avb.2022.101762

[B6] Bernstein C. Trimm L. (2016). The impact of workplace bullying on individual wellbeing: the moderating role of coping. *SA J. Hum. Resource Manag.* 14 1–12. 10.4102/sajhrm.v14i1.792

[B7] Brito C. C. Oliveira M. T. (2013). Bullying and self-esteem in adolescents from public schools. *J. Pediatr.* 89 601–607. 10.1016/j.jped.2013.04.001 24029549

[B8] Cong C. W. Ling W. S. Aun T. S. (2021). Problem-focused coping and depression among adolescents: mediating effect of self-esteem. *Curr. Psychol.* 40 5587–5594. 10.1007/s12144-019-00522-4

[B9] de Onis M. Onyango A. W. Borghi E. Siyam A. Nishida C. Siekmann J. (2007). Development of a WHO growth reference for school-aged children and adolescents. *Bull. World Health Organ.* 85 660–667. 10.2471/BLT.07.043497 18026621 PMC2636412

[B10] Doi S. Fujiwara T. Isumi A. (2020). Association between maternal adverse childhood experiences and child’s self-rated academic performance: results from the K-CHILD study. *Child Abuse Negl.* 104:104478. 10.1016/j.chiabu.2020.104478 32247070

[B11] Folkman S. Lazarus R. S. (1980). An analysis of coping in a middle-aged community sample. *J. Health Soc. Behav.* 21 219–239. 10.2307/2136617 7410799

[B12] Fujiwara T. Doi S. Isumi A. Ochi M. (2020). Association of existence of third places and role model on suicide risk among adolescent in japan: results from A-CHILD study. *Front. Psychiatry* 11:529818. 10.3389/fpsyt.2020.529818 33192648 PMC7644899

[B13] Hamamura T. (2012). Are cultures becoming individualistic? a cross-temporal comparison of individualism-collectivism in the United States and Japan. *Pers. Soc. Psychol. Rev.* 16 3–24. 10.1177/1088868311411587 21700795

[B14] Heine S. J. Hamamura T. (2007). In search of East Asian self-enhancement. *Pers. Soc. Psychol. Rev.* 11 4–27. 10.1177/1088868306294587 18453453

[B15] Heine S. J. Lehman D. R. Markus H. R. Kitayama S. (1999). Is there a universal need for positive self-regard? *Psychol. Rev.* 106 766–794. 10.1037/0033-295X.106.4.766 10560328

[B16] Keane L. Loades M. (2017). Review: low self-esteem and internalizing disorders in young people - a systematic review. *Child Adolesc. Ment. Health* 22 4–15. 10.1111/camh.12204 32680408

[B17] Kobayashi E. Farrington D. P. (2020). Why do japanese bully more than americans? influence of external locus of control and student attitudes toward bullying. *Educ. Sci. Theory Practice* 20 5–19. 10.12738/jestp.2020.1.002

[B18] Lodge J. Feldman S. S. (2007). Avoidant coping as a mediator between appearance-related victimization and self-esteem in young Australian adolescents. *Br. J. Dev. Psychol.* 25 633–642. 10.1348/026151007X185310

[B19] Machmutow K. Perren S. Sticca F. Alsaker F. D. (2012). Peer victimisation and depressive symptoms: can specific coping strategies buffer the negative impact of cybervictimisation? *Emot. Behav. Difficulties* 17 403–420. 10.1080/13632752.2012.704310

[B20] Manani P. Sharma S. (2016). Self esteem and suicidal ideation: a correlational study. *MIER J. Educ. Stud. Trends Practices* 3 75–83. 10.52634/mier/2013/v3/i1/1556

[B21] McGee R. Williams S. Nada-Raja S. (2001). Low self-esteem and hopelessness in childhood and suicidal ideation in early adulthood. *J. Abnorm. Child Psychol.* 29 281–291. 10.1023/A:1010353711369 11523834

[B22] Mello Z. R. (2019). *A Construct Matures: Time Perspective’s Multidimensional, Developmental, and Modifiable Qualities.* Taylor & Francis: Milton Park. 10.1080/15427609.2019.1651156 PMC7540185 33033466

[B23] Mishna F. Khoury-Kassabri M. Schwan K. Wiener J. Craig W. Beran T. (2016). The contribution of social support to children and adolescents’ self-perception: the mediating role of bullying victimization. *Children Youth Services Rev.* 63 120–127. 10.1016/j.childyouth.2016.02.013

[B24] Moon J. Mello Z. R. (2021). Time among the taunted: the moderating effect of time perspective on bullying victimization and self-esteem in adolescents. *J. Adolescence* 89 170–182. 10.1016/j.adolescence.2021.05.002 34020289

[B25] Nagamitsu S. Mimaki M. Koyanagi K. Tokita N. Kobayashi Y. Hattori R. (2020). Prevalence and associated factors of suicidality in Japanese adolescents: results from a population-based questionnaire survey. *BMC Pediatr.* 20:467. 10.1186/s12887-020-02362-9 33023527 PMC7542337

[B26] Olweus D. (1993). *Bullying at School: What we Know and what we can do (7^a^; Reimpresión)”.* Oxford: Blackwell.

[B27] Otake K. Shimai S. Soga S. (2001). Coping scale brief version elementary school children health. *Hum. Sci.* 4 1–5.

[B28] Overholser J. C. Adams D. M. Lehnert K. L. Brinkman D. C. (1995). Self-esteem deficits and suicidal tendencies among adolescents. *J. Am. Acad. Child Adolesc. Psychiatry* 34 919–928. 10.1097/00004583-199507000-00016 7649963

[B29] Sakurai S. (1992). The investigation of self-consciousness in the 5th-and 6th-grade children. *Japanese J. Exp. Soc. Psychol.* 32 85–94. 10.2130/jjesp.32.85 28548501

[B30] Silva G. Lima M. L. C. Acioli R. M. L. Barreira A. K. (2020). Prevalence and factors associated with bullying: differences between the roles of bullies and victims of bullying. *J. Pediatr.* 96 693–701. 10.1016/j.jped.2019.09.005 31707041 PMC9432314

[B31] Smith L. Shin J. I. Carmichael C. Oh H. Jacob L. López Sánchez G. F. (2021). Prevalence and correlates of multiple suicide attempts among adolescents aged 12-15 years from 61 countries in Africa, Asia, and the Americas. *J. Psychiatr. Res.* 144 45–53. 10.1016/j.jpsychires.2021.09.047 34598008

[B32] Sowislo J. F. Orth U. (2013). Does low self-esteem predict depression and anxiety? a meta-analysis of longitudinal studies. *Psychol. Bull.* 139 213–240. 10.1037/a0028931 22730921

[B33] Swearer S. M. Hymel S. (2015). Understanding the psychology of bullying: moving toward a social-ecological diathesis–stress model. *Am. Psychol.* 70:344. 10.1037/a0038929 25961315

[B34] Trompeter N. Bussey K. Fitzpatrick S. (2018). Cyber victimization and internalizing difficulties: the mediating roles of coping self-efficacy and emotion dysregulation. *J. Abnorm. Child Psychol.* 46 1129–1139. 10.1007/s10802-017-0378-2 29238887

[B35] van Geel M. Goemans A. Zwaanswijk W. Gini G. Vedder P. (2018). Does peer victimization predict low self-esteem, or does low self-esteem predict peer victimization? meta-analyses longitudinal studies. *Dev. Rev.* 49 31–40. 10.1016/j.dr.2018.07.001

[B36] Zhong M. Huang X. Huebner E. S. Tian L. (2021). Association between bullying victimization and depressive symptoms in children: the mediating role of self-esteem. *J. Affect. Disord.* 294 322–328. 10.1016/j.jad.2021.07.016 34311332

[B37] Zhou Z.-K. Liu Q.-Q. Niu G.-F. Sun X.-J. Fan C.-Y. (2017). Bullying victimization and depression in Chinese children: a moderated mediation model of resilience and mindfulness. *Personal. Individual Differ.* 104 137–142. 10.1016/j.paid.2016.07.040

[B38] Zimbardo P. G. Boyd J. N. (2015). “Putting time in perspective: a valid, reliable individual-differences metric,” in *Time Perspective Theory; Review, Research and Application: Essays in Honor of Philip*, eds Stolarski M. Fieulaine N. van Beek W. (Berlin: Springer), 17–55. 10.1007/978-3-319-07368-2_2

